# Targeting immuno-oncology metabolism for precision cancer therapy

**DOI:** 10.3389/fonc.2023.1124715

**Published:** 2023-02-02

**Authors:** Sakshi Pajai, Jyoti E. John, Satyendra Chandra Tripathi

**Affiliations:** Department of Biochemistry, All India Institute of Medical Sciences, Nagpur, MH, India

**Keywords:** cancer, metabolism, therapeutics, metabolic reprogramming, tumour immunology, immune cells, immuno-oncology

## Abstract

Immune cells play a key role in host defence against infection and cancer. Unlike infection, cancer is a multidimensional disease where cancer cells require continuous activation of certain pathways to sustain their growth and survival. The tumour milieu plays an important role in defining the metabolic reprogramming to support this growth and evasion from the immune system. Cancer and stromal cells modulate each other’s metabolism during cancer progression or regression. The mechanism related to change in the metabolism and its role in the crosstalk between tumour and immune cells is still an area of immense importance. Current treatment modalities can be immensely complemented and benefited by targeting the immuno-oncology metabolism, that can improve patient prognosis. This emerging aspect of immune-oncology metabolism is reviewed here, discussing therapeutic possibilities within various metabolic pathways and their effect on immune and cancer cell metabolism.

## Introduction

It is well recognized that cancer cells can alter and rewire cellular signaling networks to adapt to stress conditions and continue to proliferate (1). Being a metabolically heterogeneous disease and cancer can promote the metabolic plasticity which can impact tumorigenicity (2). Reprogramming, which is considered as a hallmark of transformation, may be necessary at the genetic and epigenetic levels to maintain the malignant transformation. The tumour microenvironment lacks enough supply of oxygen and nutrients due to its usage by cancer cells (3). Immune metabolism, which is an interplay between the tumour, its microenvironment and immune cells, aims at studying the immune system and body’s metabolic functions in a broad perspective. Previous studies have demonstrated that the interaction and cooperative action of cells in the tumour microenvironment alter the tumour and immune cell metabolism and promote malignancy (4–6). The extracellular matrix proteins and other soluble factors in the tumour microenvironment, standardize heterogeneity and clonal evolution and facilitate cancer cell proliferation and, inevitably, metastasis. The majority of tumour types are characterized by a microenvironment that is nutrient-poor, hypoxic, and acidic because of high levels of lactate, produced due to anaerobic glycolysis (7). Extracellular lactate concentrations are correlated with tumour vascularization, systemic immune suppression, and tumour progression. Similarly, it has been found that adenosine also modifies tumour microenvironment to make it favorable for cancer cells by stimulating anti-inflammatory activities, which hampers the sustainability of immune cells. The interaction of innate and adaptive immunity can drive a well-regulated and effective response against tumorigenesis. The tumour microenvironment is infested by innate immune cells such macrophages, dendritic cells, and natural killer cells as well as T cells and B cells of adaptive immunity. T cells engage directly with cancer cells as well as communicate and activate various cellular components in tumour milieu. The B cells have antitumor and protumor functions that are yet to be discovered. On the other hand, depending on the stimulus provided within the tumour microenvironment, macrophages can be categorized as pro-inflammatory (M1) or anti-inflammatory (M2). While IL-4 and IL-1 induce the M2 phenotype, interferon (IFN) and toll-like receptor (TLR) ligands favor the M1 phenotype (8). There has always been partial success in accessing the therapeutic modalities again as metabolic switching is not taken into account.

Immune responses can both destroy tumour cells as well as help accelerate tumour progression through selective pressures created in the dynamic and constantly changing interactions between the immune system and cancer cells (9). The interaction of immune system with cancer is thought to progress through at least three steps, which are known as elimination, equilibrium, and escape. Newly formed cancer cells get destroyed by the immune system during the elimination phase, which occurs frequently during the early stages of carcinogenesis. No clinically detectable tumours form if the elimination stage is successful in identifying all pre-malignant and malignant cells. On the other hand, failure to totally eliminate cancer cells may lead to an equilibrium phase in which the immune system can suppress the tumour growth but is unable to destroy it. This stage involves some immune evasion, that can put cancer cells under strong selective pressure to undergo mutation that would enable them to evade immune surveillance, either by compromising their immunogenicity or by establishing a localized immunosuppressive environment. Successful completion of these processes leads to the escape phase, which permits uncontrollable cancer cell growth and the development of clinically evident tumours with different levels of immune evasion and suppression (10). Cancer cells’ immune evasion and metabolic reprogramming are interdependent as the cancer cells modulate the tumour milieu to establish the metabolic intermediates involved in suppressing the immune responses (11). The role of immune-onco-metabolism is now well recognized; however, further research and insights are still needed to establish the role and its impact on cancer progression. Immunotherapy-based treatments have shown remarkable success in recent years, with cytokine treatments, therapeutic vaccines, immune checkpoint inhibitors (ICIs), and small molecules being recent examples of active immunotherapies (12). In this review, we discuss the interaction between the cells of tumour milieu and cancer cells with possible therapeutic opportunities related to these interactions.

## Modulation of immuno-oncology metabolism through carbohydrate metabolism

**Figure 1:**
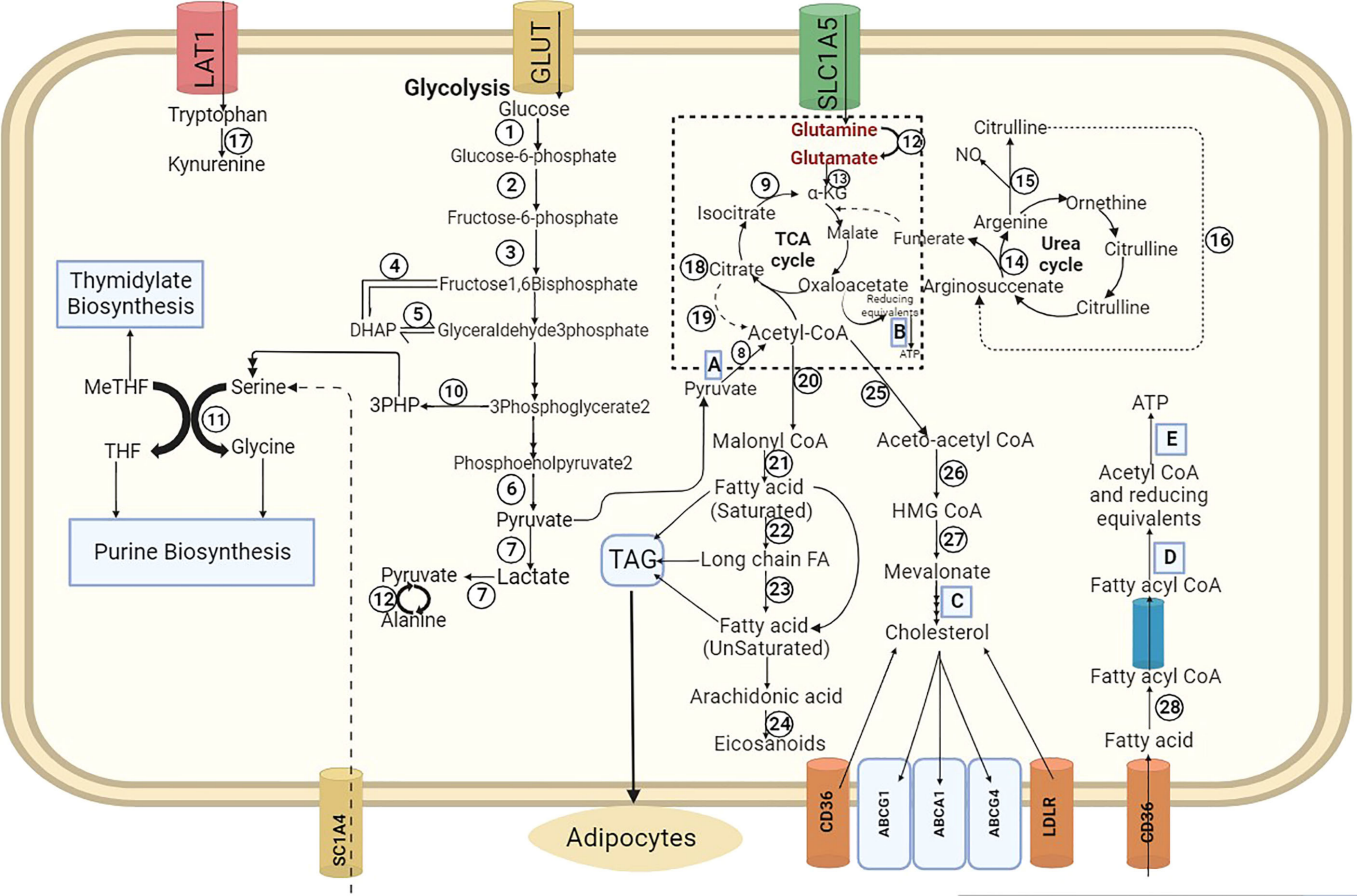
Overview of Carbohydrate, Amino acid, Fatty acid metabolism-hexokinase, phosphor-glucose isomerase, phosphofructokinase, aldolase, triose phosphate isomerase, pyruvate kinase, lactate dehydrogenase, pyruvate dehydrogenase, isocitrate dehydrogenase, phosphoglycerate dehydrogenase, serine hydroxymethyl transferase, transaminase, glutamate dehydrogenase, arginosuccinate lyase, nitric oxide synthase, arginosuccinate synthase, IDO/TDO, citrate synthase, ATP citrate lyase, acetyl-CoA carboxylase, fatty acid synthase, elongase, desaturase, cyclooxygenase (1 and 2), acetyl-CoA acyl transfers, HMG CoA synthase, HMG CoA reductase, fatty acyl CoA synthase. **(A)** mitochondrial pyruvate carrier, **(B)** electron transport chain, **(C)** cholesterol synthesis, **(D)** β-Oxidation, **(E)** oxidative phosphorylation.

Glycolysis, which involves the breakdown of glucose into pyruvate remains the main metabolic pathway of the carbohydrate mechanism. The expedient byproducts include glucose-6-phosphate, which can be used for NADPH production and synthesis of a pentose sugar, ribose through the pentose phosphate pathway (PPP), ribose-5-phosphate which is the main precursor of nucleic acid synthesis (13). The conversion of end product pyruvate to acetyl-CoA is another important aspect, recognizing its participation in the TCA cycle (**Figure 1**) (10). Cancer cells and other cells that proliferate rapidly consume a lot of glucose and produce a lot of lactate (14). p53, c-Myc, and hypoxia-inducible factor (HIF1) are a few oncogenes and growth factors that regulate glucose metabolism in cancer cells. This metabolic reprogramming involves Notch, Akt, phosphoinositide-3-kinase (PI3K), AMP-activated protein kinase (AMPK), and mammalian target of rapamycin (mTOR). C-Myc can stimulate mitochondrial biogenesis in cancer cells, resulting in an increase in the number of mitochondria and inducing enzymes involved in the glycolytic pathway (15). During tumour metabolic reprogramming, p53 regulates glycolysis by inhibiting the glucose transporters GLUT-1 and GLUT-4 and inducing the TP53-inducible glycolysis and apoptosis regulator (TIGAR). In response to apoptosis, p53 can inhibit the same pathway whereas c-Myc can initiate glutaminolysis (16). Increased fructose levels enhance the accumulation of inflammatory cytokines, whereas galactose has been shown to influence immune cell functioning in the tumour microenvironment (17).

Immune effector cells, such as activated cytotoxic T cells, target and destroy cancer cells while undergoing self-metabolic reprogramming required to carry out cancer elimination functions (18). T cells regulate the glucose transporter GLUT-1 to promote glucose absorption and glycolysis during antigenic stimulation (19) However, anaerobic glycolysis is necessary for T cells to carry out effector functions, and this cannot be sustained in a permanent state. As a result, memory T cells, which mostly depend on mitochondrial respiration rather than aerobic glycolysis, increases during acute infection immediately after briefly increasing the effector T cells. Contrarily, cancer cells consume an excessive amount of glucose due to a high proliferation rate, hence limiting the glucose availability for the other cells in the tumour microenvironment (20). Utilizing glucose in accordance with their active state is the primary characteristic of T cells. For this, naive T cells require glucose to generate ATP through the OXPHOS and tricarboxylic acid (TCA) cycle whereas activated T cells do conversion using OXPHOS cycle into aerobic glycolysis. B cells are not that momentous in the utilization of glucose as it depends on the stage of B cell development. However, in a hypoxic environment, the germinal centres of B cells exhibit increased glycolytic activity. NK cells have not been studied in detail but it has been demonstrated that NK cells utilize glucose *via* glycolysis and OXPHOS (21). It is demonstrated that M1-like macrophages necessitate a high glycolytic activity while the M2-like macrophages depend on the OXPHOS (22). Macrophages have also been shown to accelerate the PPP through the modulation of CARKL to assist the increasing demand for NADPH (23). Dendritic cells do not have much significance except for the fact that they have been known to maintain immune function by consuming the stored glycogen.

## Targeting carbohydrate metabolism

The foremost reason for the reduction in the effector function of immune cells is due to the formation of a nutrient-deprived milieu by the cancer cells due to their dissolute metabolizing capacity. It creates an alteration from effector to regulatory functioning of the T cells by favouring the FOXP3 expression (24). The prime focus of steering cancer associate glucose metabolism is either by targeting enzymes of the glycolytic pathway or by using an analogue of glucose that can act as a competitive inhibitor e.g., 2-DG. One of the major areas of focus would be targeting immune checkpoints, like PD-1, PDL1, and CTLA-4 by using checkpoint inhibitors (25). The usage of PD-1 is broadly studied and is vaguely effective in curbing the cancer cell proliferation. All these approaches have been hugely successful in inhibition of tumours with a high neoantigen load or a high glycolytic index. There has been a build-up of lactate in the microenvironment which tends to inhibit the T cell function and its proliferation (26). However, neutralization of the environment with bicarbonate has fairly been successful so far. Mouse model studies have shown promise results on using V-domain Ig suppressor of T-cell activation (VISTA) in combination with anti-PD1 for neutralizing the microenvironment (27). The usage of a lactate dehydrogenase (LDH) is not encouraged due to its differential effects on the immune cells (28). In the bottom-line, it is essential to perform a clinical evaluation of the effect on immune cells by using these therapeutic approaches (**Table 1**).

**Table 1:** Summary of anticancer drugs targeting Carbohydrate, Amino acid and Fatty acid metabolism.

Target	Therapy	Cancer type	References
**GAPDH**	3-Bromopyruvate, ornidazole, a-chlorohydrin	Liver cancer, Lymphoma, Breast cancer	(29–31)
**GLUT**	2-Deoxyglucose, Phloretin, Silybin, Glutor, STF-31, WZB117, Fasentin, Ritonavir, 2,5-AM, Apigenin, Genistein, Cisplatin, Metformin, Tamoxifen, EGCG, Hesperetin, Kaempferol, Silybin	Breast cancer, Colorectal cancer, Lung cancer, Lymphoma, Osteosarcoma, Renal cell carcinoma, Hepatocellular cancer, Prostate cancer, Ovarian cancer, Melanoma, Multiple Myeloma	(30–32)
**HK**	2-Deoxyglucose, 3-bromopyruvate, lonidamine, methyl jasmonate, Polydatin, Tamoxifen, Metformin, EGCG	Breast cancer, colon cancer, lymphoma, neuroblastoma, pancreatic cancer, Melanoma, Hepatocellular cancer, Prostate cancer	(29–33)
**LDH**	Cetuximab, Metformin, Oxamate, Chidamide, Galloflavin, Cisplatin, 2,3-Dihydroxynaphtalen-1 Carboxylic acid, N-hydroxy-2-carboxy-substituted indoles, 3-hydroxyisoxazole-4-carboxylic acid, FK866, AZD3965, AR-C155858, Quercetin	Glioblastoma, lymphoma, pancreatic cancer, Colon cancer, Breast cancer	(31–33)
**MCT**	Quercetin, α-cyano-4-hydroxycinnamate and Lonidamine	Prostate cancer, Breast cancer, Lymphoma	(30)
**PFK**	3PO, PFK15, PFK158	Breast cancer, lymphoma, melanoma, Gastric cancer, Breast cancer	(29–31)
**PK**	TLN-232/CAP-23, Shikonin, Alkannin, TEPP-46, DASA-58, ML-265, oleanolic acid, dimethylaminomicheliolide, Orlistat, 5-FU, Lapatinib, EGCG, Quercetin, Vit K3, Vit K5	Breast cancer, glioblastoma, liver cancer, lung cancer, melanoma, renal cell carcinoma, Ovarian cancer, Bladder cancer	(29–32)
Arginine metabolism **ARG 1**	CB-1158 BEC hydrochloride nor-NOHA	Advanced and metastatic solid tumours Tumour-specific Th2 cells Breast cancer	(34, 35)
Glutamine metabolism: **GLS1** **GLS** **GLS1**	CB-839 BPTES 968	Non-small cell lung cancer, myeloma Hepatocellular carcinoma, B-cell lymphoma, Pancreatic cancer Non-small cell lung cancer	(34, 35)
**ACAT1**	Avasimibe, avasimin, and bitter-melon extract	Breast cancer, colorectal cancer, prostate cancer	(36)
**ACC**	TOFA, metformin, AICAR ND-654, and ND-646	Cervix cancer, colon cancer, head and Neck cancer, Hepatocellular carcinoma, Lung cancer, Ovarian cancer, Prostate cancer, Renal cell carcinoma	(37, 38)
**ACLY**	SB-204990 LY294002	Lung cancer	(39)
**CPT1**	Etomoxir, ranolazine, and perhexiline	Breast cancer, glioblastoma, lymphocytic leukaemia, prostate cancer	(40–42)
**FASN**	C75, cerulenin, orlistat, triclosan, EGCG, TVB-3166, and amentoflavone	Breast cancer, endometrial cancer, glioblastoma, lung cancer, melanoma, mesothelioma, ovarian cancer, prostate cancer, renal cell carcinoma	(43)
**HMGCR**	Statins and lipophilic statins	Colorectal cancer, melanoma, multiple myeloma, prostate cancer	(44)

## Modulation of immuno-oncology metabolism through amino acid metabolism

Since amino acids are the building blocks of proteins, they serve all the important nutrients that are related to immune-oncology metabolism (**Figure 1**). Glutamine, one of the most extensively studied non-essential amino acids, is known for its key role with cancer proliferation (45). Modification in glutamine metabolism has been known to have adverse effects on immune cells. It has been shown that cancer cells possess the mutated Myc gene, which can induce glutaminolysis and increase the uptake of glutamine from extracellular space. It has been observed that a reduction in levels of glutamine stimulates T_reg_ proliferation, whereas the B cells and macrophages will require a glutamine-rich environment for their multiplication (46). Arginine is another important amino acid, which helps in upregulating the immune cells *via* the Arg1 and iNOS enzymes. It is also observed that a high level of expression of Arg-1 leads to the degradation of arginine by the M2 macrophages, leading to a negotiated antigen-specific T-cell response. The role of serine and glycine is not widely known or studied, but it is known that purine biosynthesis requires serine, essential for T-cell multiplication (47). Tryptophan which is an essential amino acid is believed to play an important role in cancer biology, especially in the kynurenine pathway. Increased production of kynurenine is said to lessen T-cell expression. Low to moderate levels of tryptophan have been shown to impair Th17 function and promote Treg development (48). As a crucial component of tumour metabolism, the exchange of metabolites between ferroptosis and cancer cells is becoming widely accepted. There is evidence that amino acid (AA) metabolism is necessary for ferroptosis (49). Various experimental cancer models have shown that ferroptosis inducers, that target ferroptosis-suppressor-protein 1 (FSP1) and glutathione peroxidase 4 (GPX4), deplete glutathione (GSH), or enhance the iron pool, can effectively kill cancer cells, especially drug-resistant cancer cells (50). Cysteine can be catalyzed by glutamate-cysteine ligase (GCL) to produce the antioxidant GSH. Cysteine can also inhibit ferroptosis independently of GSH by triggering the Rag-mTORC1-4EBPs signaling axis and improving the production of the protein GPX4. Cysteine is added to cystine-glutamate antiporter transport system (System Xc-) in order to simultaneously generate GSH and exchange Glu. By promoting the production of glutamine peptide, the glutamate-cysteine ligase catalytic subunit (GCLC) prevents ferroptosis and preserves the equilibrium of the glutamate pool under cystine deprivation (51, 52). Tryptophan (Trp) can be transformed into N-formylkynurenine by indoleamine-2,3-dioxygenase (IDO) and TDO under catalysis to regulate the growth of tumour. IDO blocks xCT (solute carrier family 7 member 11), aggravating ferroptosis. In order to prevent ferroptosis, Trp can also be converted to indole-3-pyruvate through the catalysis of IL4i1(interleukin 4 induction 1) (53).

## Targeting amino acid metabolism

The core tactic in using amino acid metabolism for therapeutic purposes is to dispossess the metabolites or impede the main regulatory enzymes. As an example, inhibition of GLS (Glutaminase) tends to persuade mitochondrial stress and thus diminution the glycolytic activity in the cancer cells. Studies in breast cancer models have shown that inhibition of GLS has supported the proliferation of M1 macrophages and reduced tumours (54). GLS inhibition can also regulate effector function and survival of T cells. Whilst studies are being done on arginine starvation which shows tremendous potential in maintaining an immune suppressive microenvironment with low side effects. Inhibition of Gln conversion to Glu or its uptake using compounds like BPTES and Tamoxifen respectively can also trigger ferroptosis for cancer treatment and to target drug resistant cells (49, 50). Targeting Arg1 in the acidic tumour microenvironment can cause decreased levels of tumour growth factors and can improve T effector functions leading to tumour regression (**Table 1**). Similarly, inhibition of the enzymes in the tryptophan metabolism (IDO, TDO) have shown immense potential in pre-clinical analysis but produced miserable results in clinical trials. Interestingly, IDO inhibitors showed better outcomes when combined with conventional treatment modalities such as chemotherapy, radiotherapy, and immunotherapy. The biggest success came with the usage of the dendritic cell vaccine along with the IDO inhibitors, which can convert the Treg cells to Th17 phenotype and support cytotoxic T cells mediated destruction of the cancer cells (55). Targeting indole-3-pyruvate (I3P), a Trp metabolite, which traps lipid peroxyl radicals and prevent ferroptosis is another key strategy to target tumour mass (53). Induction of cell ferroptosis through targeting the Xc-system-mediated absorption of cystine is a classic strategy in fundamental research. Drugs such as sorafenib, erastin and sulfasalazine are in clinical trials, which prevent cysteine accumulation and hence deplete GSH to enhance ferroptosis. Cystinase targets cysteine and hence induces depletion of cystine. The iron-starvation response, which is induced by NFS1 and has been demonstrated to protect cells against ferroptosis. Hence, targeting NFS1 can be a viable strategy for to induce ferroptosis in cancer cells (49, 51, 52).

## Modulation of immuno-oncology metabolism through lipid metabolism

Just as carbohydrates, lipids are also instrumental in energy storage functions and are an important constituent of various signaling pathways (**Figure 1**). The biggest supportive factor about the association of cancer with lipids came with obesity, wherein studies showed that obesity promotes a 1.5 times high risk of cancer (56). *De novo* fatty synthesis is known to curb the damage induced by reactive oxygen species generated by deranged metabolic pathways in cancer cells. The major lipid pathways congregate around Acetyl-CoA, which acts as a central player in metabolism due to its convergence with various metabolic pathways. Cholesterol is again one of the main lipids whose synthesis pathway is transcriptionally regulated by sterol regulatory element-binding protein (SREBP). All of these form potential targets for immune-oncology metabolism because of their active involvement in various metabolic functions. The mevalonate pathway regulates ferroptosis by affecting selenoprotein biosynthesis (57). Ferroptosis is also being regulated by fatty acid pools in cancer cells, which is modulated by stromal and immune cells in TME. Immune cells can affect the iron, lactate, and lipid metabolism which in turn can modulate ferroptosis in cancer cells (58). Lipid metabolism is also highly correlated to the sensitivity of cells to ferroptosis. Lysophosphatidyl-choline acyltransferase 3 (LPCAT3) and Acyl-CoA Synthetase Long Chain Family Member 4 (ACSL4) can incorporate polyunsaturated fatty acids (PUFA) into the membrane. Either an enzymatic catalysis or a non-enzymatic free radical chain reaction can be used to oxidise PUFA (59). The build-up of lipid peroxides, particularly phospholipid peroxides, is thought to represent ferroptosis during this period. Currently, it is generally accepted that lipid peroxides are the primary cause of ferroptosis. When an excessive amount of lipid peroxides accumulates, this damage to the plasma membrane eventually results in the occurrence of ferroptosis in the cells (60).

## Targeting fatty acid metabolism

Since cancer cells form a storehouse of many key regulatory enzymes involved in lipid metabolism, targeting their inhibition could be of potential use. One such example is the (Fatty Acid Synthase) FASN enzyme which is highly expressed in cancer cells and is also considered to be a marker for poor prognosis of cancer. FASN has been explored as a target by the use of cerulenin, C75, IPI-9119 and orlistat as its inhibitors (61). Similarly, rate-limiting enzymes such as ATP citrate lyase (ACLY) and Acetyl-CoA can also be putatively targeted. Inhibition of these enzymes by using inhibitors like ND-654 and SB-204900 has shown proficiency in many tumours. Lipids when gets stored in the cells have been shown to weaken the antigen presentation. However, since the studies are done *in-vitro* and in murine models, the benefits are only postulated. The cancer cells have also been shown to increase their requirement for fatty acids (62). Since cellular cholesterol uptake is mediated by LDL receptors and the cluster of differentiation 36 (CD36) protein, these molecules are also key accessible targets. LDL receptors have been linked to both a better prognosis and survival in small-cell lung cancer, while their loss has been linked to a poor prognosis in colorectal cancer. However, there are instances of negative side effects when these receptors are targeted for bladder, renal, and pancreatic tumours. Cancers that express CD-36 have a higher propensity to metastasize. Furthermore, CD36 attenuation may be targeted to reduce tumour migration (63). Additionally, CD36 is known to support Treg activity and survival within the tumour microenvironment. Inhibition of the fatty acid-binding protein (FABP5) can control the function of T_reg_ cells and inhibit the accumulation of these cells. The anti-tumour efficacy can be increased by inhibition of fatty acid oxidation as well. Studies on inhibitors such as CPT1 and ST1326 has highly been successful for increasing the immune efficacy by blocking fatty acid oxidation (**Table 1**). Lastly, an important area is of targeting cholesterol metabolism, which has been highly successful in blocking cancer cell multiplication. Research has been done on statins to inhibit the HMGCR, a rate-limiting enzyme of the mevalonate pathway, and increase survival in cancer patients. HMGCR inhibition also leads to induction of ferroptosis by inactivating GPX4 (64). Ferroptosis can also be induced by inhibiting the GPX4-HSPA5 pathway cancer cells and has been shown to complement gemcitabine in the cancer treatment (65). There have been efforts to attenuate monoacylglycerol lipase (MAG-L) by the usage of inhibitors which have shown promise in melanoma and ovarian cancer. CD8+ T cells downregulates SLC7A11 and SLC3A2 by releasing IFN-γ, which induces ferroptosis in cancer cells, but also increases PD-L1 expression (66, 67). Hence, a combination therapy including immune checkpoint inhibitors and ferroptosis inducers can be of immense potential. The best results have come by the combination of targeting the esterification of ACAT-1 and anti-PD1 immunotherapy or chemotherapeutic agents, which have shown better efficacy than monotherapies. Hence, inhibition of rate-limiting enzymes of lipid metabolism can pave way for the regression and treatment of tumours.

## Conclusion

The aforementioned studies reveal a flexible interaction and relationship between cancer cells and immune cell metabolism. Cancer cells and stromal cells strongly modulate immune cell metabolism in the tumour microenvironment. Carbohydrate and amino acid metabolism together with lipids drive the expression of key genes responsible for changing phenotypes during cancer progression. Nutrient deficiency or reticence of key enzymes associated with the metabolic pathways that utilize these metabolites can result in the impairment of effector and cellular functions. A significant amount of research has been devoted to understand the regulation and role of metabolic play between immune and cancer cells; nevertheless, still many answers remain a mystery. Further studies are needed to understand the molecular signalling pathways that regulates gene expression, cellular and effector functions. In summary, immuno-oncology metabolism has a great potential to identify novel therapeutic targets and also provide mechanistic details into tumour progression. This has become an area of ​​great interest because a targeted approach for intervention in metabolic pathways has enormous potential to complement current therapies and improve treatment outcomes.

## Future prospects

Generally, for most human diseases, it is believed that prevention is of foremost importance than cure. But, ever since the therapeutics of cancer has expanded its horizons, this field of research demands continuous innovation with implications for a large scale of patients. However, there are certain challenges, primarily related to finance and resource building. But these problems could be catered to by approaching cancer therapeutics as a challenge to the community rather than to an individual. The area of cancer therapeutics will always remain competitive and demand new molecules of interest, with thousands of new targets in the pipeline. It has to be always clear that cancer isn’t always about the genes, it can be a dysregulation of the pathways as well. Targeting immune-oncological mechanism, particularly in the earliest stages of the disease before the disease shatters the immune system, widens the perspective as it corresponds to the pathways and reactions going in the human body, with a window for more research as we could dive deep into the intricacy of the biochemical reactions. Over the past decade, significant advancements have been made in immuno-oncology. It has enabled the treatment of aggressive cancers that were previously resistant to treatment with conventional anticancer therapies, as well as providing long-term survival benefits. A large percentage of new evolving targets are still in the preclinical stage. Challenges in cancer will demand colossal overseas investments, a collaboration of bright heads across the globe, and a massive amalgamation of academia and the industry with a vision of developing new tools and technologies for solving the bigger problem.

## Author contributions

SP and ST conceived and designed the review. All authors participated in manuscript redaction. SP unified the content and with ST and JJ designed the figures. ST and JJ edited the review. ST supervised the work. All authors contributed to the article and approved the submitted version.
