# Correction: Targeting immuno-oncology metabolism for precision cancer therapy

**DOI:** 10.3389/fonc.2025.1764570

**Published:** 2026-01-12

**Authors:** Sakshi Pajai, Jyoti E. John, Satyendra Chandra Tripathi

**Affiliations:** Department of Biochemistry, All India Institute of Medical Sciences, Nagpur, MH, India

**Keywords:** cancer, metabolism, therapeutics, metabolic reprogramming, tumour immunology, immune cells, immuno-oncology

The title of this article was erroneously given as: “Targeting immune-onco-metabolism for precision cancer therapy”. The correct title of the article is “Targeting immuno-oncology metabolism for precision cancer therapy”.

The original version of this article has been updated.

